# Exploring anhedonia in adolescents with Chronic Fatigue Syndrome (CFS): A mixed-methods study

**DOI:** 10.1177/13591045211005515

**Published:** 2021-04-16

**Authors:** Lucie Smith, Esther Crawley, Madeleine Riley, Megan McManus, Maria Elizabeth Loades

**Affiliations:** 1 Department of Psychology, University of Bath, Bath, UK; 2 Bristol Medical School, University of Bristol, Bristol, UK; 3 University of Edinburgh, Edinburgh, UK

**Keywords:** Chronic fatigue syndrome, mood disorder, qualitative, survey

## Abstract

**Background:**

Chronic Fatigue Syndrome (CFS/ME) may get in the way of enjoying activities. A substantial minority of adolescents with CFS/ME are depressed. Anhedonia is a core symptom of depression. Anhedonia in adolescents with CFS/ME has not been previously investigated.

**Method:**

One hundred and sixty-four adolescents, age 12 to 18, with CFS/ME completed a diagnostic interview (K-SADS) and questionnaires (HADS, RCADS). We used a mixed-methods approach to explore the experience of anhedonia and examine how common it is, comparing those with clinically significant anhedonia to those without.

**Results:**

Forty-two percent of adolescents with CFS/ME reported subclinical or clinical levels of anhedonia. Fifteen percent had clinically significant anhedonia. Thematic analysis generated two themes: (1) stopping activities that they previously enjoyed and (2) CFS/ME obstructs enjoyment. Most (72%) of those who reported clinically significant anhedonia met the depression diagnostic criteria. Those who were depressed used more negative language to describe their experience of activities than in those who were not depressed, although the themes were broadly similar.

**Conclusions:**

Experiencing pleasure from activities may be affected in CFS/ME, particularly in those who are depressed. Anhedonia may get in the way of behavioural strategies used within CFS/ME treatments.

Chronic fatigue syndrome, or myalgic encephalomyelitis (CFS/ME) can be defined as ‘generalised fatigue persisting after routine tests and investigations have failed to identify an obvious underlying cause’ (National Institute for Health and Care Excellence [NICE], 2007). The key features include post-exertional malaise, cognitive difficulties, sleep disturbance and pain. The prevalence of CFS/ME in adolescents is between 0.4% and 2.4% (Chalder et al., 2003; Crawley et al., 2012; Rimes et al., 2007) and it significantly impacts the quality of life of affected adolescents (Winger et al., 2015). CFS/ME was found to be more disabling than juvenile idiopathic arthritis and emotional disorders in children and adolescents, specifically in school absence and illness anxiety (Garralda & Rangel, 2004). In a clinical cohort, 62% of adolescents with CFS/ME were attending school less than 40% of the time; only 11% attended full-time school and 27% attended at least 40% of the time (Crawley & Sterne, 2009).

Children and adolescents with CFS/ME are at a higher risk of developing emotional difficulties and mental health disorders compared to healthy peers. For example, a qualitative study by Winger et al. (2014) found that school absence (and therefore lack of social communication with friends) led to feelings of ‘being forgotten’ and ‘alienated’. Nearly 30% of adolescents with CFS/ME have comorbid depression, which is ten times higher than depression prevalence in the general population (Bould et al., 2013). Despite this, our systematic review found a lack of evidence for effective treatments for adolescents with co-morbid CFS/ME and depression, and limited understanding of how depression might influence outcomes of CFS/ME (Loades et al., 2016). Further research is needed to understand the experiences of adolescents with this co-morbidity to enhance treatment for this population and understand the impacts of this comorbidity on recovery from CFS/ME.

Anhedonia is a core symptom of major depressive disorder and is defined as ‘markedly diminished interest and pleasure in all, or almost all of activities most of the day, or nearly every day’ (American Psychological Association [APA], 2013). Anhedonia is a broad construct with many intertwining facets (Berridge & Kringelbach, 2011; Rømer Thomson, 2015), including the distinction between anticipatory pleasure (i.e. the experience of excitement preceding a pleasurable activity) and consummatory pleasure (i.e. the experience of enjoyment during and following the pleasurable activity). The DSM-5 discriminates between loss of pleasure and loss of interest as components of anhedonia – the former being loss of enjoyment during activities and the latter being lack of initial motivation to engage in formerly enjoyable activities (Shankman et al., 2014). Anhedonia can also be conceptualised in a wider dimensional perspective as a general loss of positive affect. Positive affect provides a source of motivation and affective reward in goal-directed behaviour, deficits of which are present in those with anhedonia (De Fruyt et al., 2020). Anhedonia is common in adolescents with depression, occurring in over 50% of diagnoses in a clinical cohort (Goodyer et al., 2017; Orchard et al., 2017). Importantly, those who experience anhedonia have poorer treatment outcomes (McMakin et al., 2012). A recent qualitative study investigated experiences of anhedonia in a sample of adolescents either seeking treatment from a clinical service with a primary diagnosis of depression or were recruited from the community with elevated symptoms of depression. Reported themes included of loss of positive emotion, motivation and willingness to exert effort, dissociation and social withdrawal (Watson et al., 2020). Further understanding of how common anhedonia is as a symptom of depression and how it is experienced in this population could therefore give insight into how to effectively treat those who have co-morbid CFS/ME and depression.

Possibly due to the disabling impact of CFS/ME on adolescents, those who are depressed may be particularly likely to present with anhedonia as part of the depressive symptom profile. However, no previous studies have detailed how common anhedonia is as a symptom of depression in adolescents with co-morbid CFS/ME and depression. A qualitative study investigating the experience of depression for adolescents with CFS/ME found that activity restrictions resulting from fatigue were perceived to precede feelings of low mood and frustration (Taylor et al., 2017). Low mood then impacted on adolescents’ motivation to engage in activities. Negative appraisals concerning their restrictions due to CFS/ME and its symptoms hindered the enjoyment of activities when they were able to participate. This suggests that adolescents might be particularly inclined to experience anhedonia, due to their restricted activity and the accompanying negative emotions that reinforce the lack of motivation and interest.

Our study sought to quantify how common anhedonia is and to explore the experience of anhedonia in adolescents with CFS/ME. Furthermore, we aimed to compare those who met the criteria for a diagnosis of depression (henceforth referred to as ‘depressed’) to those who did not (‘not depressed’).

## Methods

### Design

This is a mixed methods cross-sectional study in a clinical cohort.

### Participants

Participants were recruited from a specialist paediatric CFS/ME service in the South West of England as part of a study investigating wellbeing in adolescents with CFS/ME. Adolescents, aged 12 to 18, with a confirmed diagnosis of CFS/ME (NICE, 2007) at their first appointment with the service were invited to take part. Participants were not eligible to participate if they were too severely affected by their illness that they were unable to take part (i.e. could not complete questionnaires or interview, even in stages over several days), nor if they were unable to complete questionnaires/an interview in English, due to budgetary constraints and issues with validity and reliability that arise when these are translated.

Through the course of the study (September 2016–February 2019), 214 patients gave consent to be contacted. Of those, 164 were interviewed, 78 declined prior to consent and 21 consented and later withdrew. Of the total 164 participants, 145 were audio recorded (88.4%).

### Measures and materials

The Kiddie Schedule for Affective Disorders and Schizophrenia (K-SADS) (Kaufman et al., 2013) is a semi-structured psychiatric diagnostic interview for mental health diagnoses in children and young people, age 6 to 18. Participants completed the following sections: major depressive disorder (containing the anhedonia section), panic disorder, agoraphobia, separation anxiety disorder, social anxiety disorder, phobic disorders, generalised anxiety disorder, obsessive compulsive disorder, eating disorders, substance use disorders and post-traumatic stress disorder. Each section presents a list of questions however gives the interviewer the opportunity to ask for further information. Responses are coded by the researcher administering the interview on a scale from 1 to 3 (1 = not present, 2 = subthreshold, 3 = threshold). Any responses meeting threshold prompts the interviewer to ask further questions to ascertain whether the individual meets the full diagnostic criteria for the relevant diagnosis. Examples of questions asked in the anhedonia section include:

Has there ever been a time you felt bored a lot of the time? When?

Did you feel bored when you thought about the things you usually like to do for fun?

Did you look forward to doing the things you used to enjoy?

Did you have to push yourself to do your favourite activities?

Interviews were completed by trained research assistants who were closely supervised graduate and undergraduate Psychology students (*N* = 7). All diagnostic decisions were made during supervision with the lead researcher, an experienced clinical psychologist.

For the purposes of this study, excerpts from the anhedonia section of the K-SADS interview were transcribed and coded using thematic analysis (Braun & Clarke, 2006).

The following questionnaires were completed as part of the clinical assessments:

The Chalder Fatigue Questionnaire or CFQ (Chalder et al., 1993) is made up of 11 items and assesses the severity of fatigue. Each item is rated on a 4-point Likert scale. Scores range from 0 to 33, and higher scores indicate higher fatigue. The CFQ has been validated for use in adults with CFS (Cella & Chalder, 2010).

The Short Form 36 Physical Functioning Subscale or SF36PFS (McHorney et al., 1993; Ware & Sherbourne, 1992) consists of 10 items which are designed to capture physical functioning by asking about limitations in tasks of daily living, such as climbing the stairs or getting dressed. Scores range from 0 to 100, and higher scores indicate better functioning. There is evidence that this measure is reliable and valid in adolescents with CFS (Loades et al., 2020b).

The Hospital Anxiety and Depression Scale or HADS (Zigmond & Snaith, 1983) is a 14 item scale, of which seven items relate to depression symptoms and seven items to anxiety symptoms. Scores range from 0 to 42, and higher scores indicate greater endorsement of symptoms. The HADS has been validated for use in adolescents (White et al., 1999).

### Procedure

Patients were introduced to the research project at their first clinical assessment appointment by their assessing healthcare professional and offered the opportunity to be contacted by a member of the research team. Potentially interested participants were contacted by a research assistant who explained the aims of the study – investigating CFS/ME and low mood – and the procedure. All interested participants and their parents completed an online or pen-and-paper consent form. The K-SADS diagnostic interview was administered by a trained research assistant, either in person (*N* = 23, 14.0%), via Skype (*N* = 48, 29.3%), or over the telephone (*N* = 89, 54.3%), according to participant preference (*N* = 4 missing data).

Participants had the choice to be interviewed either with their parents (*N* = 133, 81.1%) or separately (*N* = 31, 18.9%), were offered rest breaks throughout the interview and were given the option of completing the interview over several sessions. The anhedonia section of the interviews of all participants who scored 3 (threshold), indicating that the interview rated them as having clinically significant anhedonia, was transcribed.

The NHS Health Research Authority (16/SW/036 and (15/SW/0124) and the Psychology Research Ethics Committee at the University of Bath (16-203) granted ethical approval for this study.

### Data analysis

#### Quantitative data

Descriptive analyses were conducted to gain estimates of the prevalence of anhedonia for study participants. Group comparisons using independent samples *T*-tests were conducted to compare the functioning and fatigue of those who scored threshold for anhedonia from those who did not score threshold for anhedonia.

#### Qualitative data

The anhedonia sections of the K-SADS interviews for those participants who scored threshold for anhedonia (*N* = 25) were transcribed between April and June 2019. A reflexive thematic analysis (Braun & Clarke, 2006, 2019) was conducted using solely the transcriptions of participants who met threshold criteria for anhedonia. Thematic analysis allows for an exploration of experience using a flexible approach. We adopted a critical realist, post-positivist approach which assumes that the reality of participants can be measured and observed, however they are not wholly aware of all the elements which shape their experience (Guba & Lincoln, 1994). As this is the first study investigating anhedonia and CFS/ME, the process was inductive. Codes were identified across the whole dataset and prevalence was determined at the level of the data item. The themes were identified at a semantic level.

Two members of the research team independently read and re-read the transcripts systematically and identified codes. Initially, codes were highlighted on each transcript and data extracts were collated in a spreadsheet for each code. These were later discussed together in terms of how codes combined to generate themes and the relationship between different codes that served as subsequent themes and subthemes. This process was iterative and transcripts were re-read many times before both researchers agreed the final themes, which were also reviewed by a third member of the research team.

## Results

During the recruitment period, 375 participants were assessed as eligible in clinic. Of these, 263 agreed to be contacted by a researcher and 185 agreed to take part in the study. The majority did not give a reason for declining participation (*N* = 40), however some clarified they had too much going on (*N* = 18), were not interested (*N* = 9), were too unwell (*N* = 6) or were uncomfortable with the interview (*N* = 5). Twenty-one participants withdrew after the consent stage with no reason provided.

One hundred and sixty-four adolescents with confirmed CFS/ME completed the K-SADS interview. Their mean age was 14.99 (*SD* = 1.50) and 115 (70.1%) were female. Participants identified their ethnicity as: White British (*N* = 146, 89.0%), Other White (*N* = 6, 3.7%), Other British (*N* = 1, 0.6%), Pakistani (*N* = 1, 0.6%). Ten participants (6.1%) did not specify their ethnicity.

Twenty-five participants (15%) met the threshold score for anhedonia on the K-SADS, indicating that the interviewer classified them as experiencing clinically significant anhedonia. A further 43 participants (27%) reported subthreshold symptoms of anhedonia. Therefore, in the total sample, 68 (42%) participants reported at least some symptoms of anhedonia. Fatigue and physical function were similar between those with and without clinical levels of anhedonia (see Table 1). Those with clinically significant anhedonia scored higher on both depression and anxiety.

**Table 1.** *T*-tests comparing equality of means for participants with clinically significant anhedonia to those without.

Measure	Mean scores (*SD*)		*t (df)*	Sig. (2-tailed)	Mean difference	Std. Error difference	95%CI of the difference	
	Participants with clinically significant anhedonia	Participants who did not have clinically significant anhedonia					Lower	Upper
Physical functioning (SF36PFS)	50.68 (23.87)	51.82 (24.16)	0.21 (149)	.838	1.14	5.56	−9.85	12.13
Fatigue (CFQ)	24.99 (4.50)	26.86 (4.04)	−1.79 (150)	.076	−1.86	1.04	−3.93	0.20
Depression* (HADS)	11.95 (4.13)	8.72 (4.16)	−3.79 (34.47)	.001	−2.61	0.84	−4.27	−0.94
Anxiety* (HADS)	10.10 (2.84)	7.49 (3.78)	−3.34 (26.77)	.003	−3.24	0.97	−5.23	−1.24

*Note*. CFQ = chalder fatigue questionnaire; SF36PFS = short form 36 physical functioning subscale; *df* = degrees of freedom; *SD* = standard deviation; CI = confidence interval.

* Equal variances not assumed.

Thematic analysis of adolescent and parent responses to the anhedonia section of the K-SADS for those who scored threshold for anhedonia (*N* = 25) for whom interviews were audio recorded (*N* = 22) generated two themes, ‘being unable to do things they previously enjoyed due to loss of interest and lack of motivation’ (with two subthemes), and ‘CFS/ME gets in the way of enjoyable activities’ (with three subthemes). Each will be presented with illustrative quotes from participants.

### Theme 1: Stopping activities that they previously enjoyed

There seemed to be two interconnected reasons why the participants didn’t do things they had previously enjoyed: loss of motivation which impacted on doing things despite being interested in them (subtheme 1) and loss of interest which meant that they didn’t want to do things (subtheme 2).

#### Loss of motivation

Participants described having lost the motivation to engage in activities they would previously have enjoyed, which they attributed to fatigue. They described feeling ‘fed up’ and frustrated.

*“Yeah, just can’t get any motivation to do writing or college work. I think it’s just because I’m more tired than usual that I just don’t want to do it.”* (P1, 16-year-old female)

Negative thoughts about the activity, such as assuming they would not enjoy the activity, prevented the participants from doing things.

*“I - it’s usually fun, it’s before I’m going out that I don’t really want to because I don’t think it’s going to be fun. . . . Not sure, I just-I just don’t feel like bothered all the time, I’d rather just stay at home sort of thing.”* (P2, 14-year-old male)

However, some did feel like they would enjoy activities, but still struggled with motivation.

*“Yeah sometimes I can’t be bothered - like I know that I’ll like it, I just keep trying”* (P3, 17-year-old female)

#### Loss of interest

Young people felt that they had lost interest in the things they used to do. Even when they had the opportunity to do things, participants would not want to. Not wanting to do activities extended beyond hobbies and interests and included small daily activities.

*“I think the more sort of day to day type of things like ‘Shall we go to the park’ or ‘Should we pop here’ or ‘Should we meet up with so-and-so’ that’s completely gone. The motivation for that*” (parent of P4, 13-year-old male)

Many participants reported completely ceasing their involvement in activities, including lower energy activities such as going out for a walk. One participant spoke about how they were reluctant to leave the home as they associated it with feelings of comfort and safety. Their fear reduced their interest in getting involved in other activities outside.

*“Um, it’s most things – yeah it’s hard to actually like want to do it.”* (P2, 14-year-old male)

*“I used to cosplay. Dressing up as characters but I have no interest or motivation to do it anymore.”* (P1, 16-year-old female)

Participants reported that they found it hard to get interested in or excited about the activity in the first place. Often, they felt that they would need to push themselves into doing activities. Feelings of boredom were frequently reported while engaging in activities which further decreased their interest.

*“Yeah, like I don’t have as much interest, like I don’t have as like, much excitement for anything anymore.”* (P6, 15-year-old female)

*“Yeah, I’m just a little bit bored with everything now.”* (P3, 17-year-old female)

### Theme 2: CFS/ME obstructs enjoyment

When they were able to overcome the hurdle of not wanting to do things, participants found that CFS/ME interfered with finding enjoyment in activities. This included how they experienced activities compared to before they got ill (subtheme 1), how CFS/ME precludes fun (subtheme 2) and how CFS/ME curtails social activities (subtheme 3).

#### Compared to before CFS/ME

Activities that participants used to do before the onset of their CFS/ME were either greatly reduced or halted completely. Many participants reported that they used to be highly active and participated in sports groups, which they are no longer able to do.

*“Yeah. I’m also used to being quite like active and stuff, like with my like um fitness and I’m just not anymore”* (P7, 14-year-old female)

“*I used to like taking my dogs out a lot-but I can’t do that anymore.*” (P8, 17-year-old female)

CFS/ME had also limited their choice of activities. Often, participants were not able to leave the house and so were restricted to sedentary activities, such as watching television.

*“It’s dif-difficult because it’s kind of limited, you can’t go out so much anymore, so you’re kind of limited to what you can do in the house sometimes”* (parent of P9, 16-year-old female)

*“I don’t really get out the house much, I’m not really able to. . .I can’t do much else [than watching TV] to be honest”* (P10, 15-year-old male)

#### Precludes fun

Participants described being unable to have fun or enjoyment from the activities they do. They described not being able to do the things they previously did for fun, and also felt that ‘new’ activities that they had taken up since developing CFS/ME were poor ‘replacement’ activities for what they used to do.

*“Yeah, just don’t really do a lot. And obviously I did loads of sports before and can’t really do them so, yeah don’t do anything for fun”* (P11, 17-year-old female)

*“Not really, I don’t really find as much enjoyment as I used to. I think it’s because they’re new things I have to do now because I can’t really do the things I used to enjoy. So I kind of feel like they’re just trying to replace things I used to be able to do.”* (P12, 16-year-old female)

When participants were able to take part in their usual activities to some degree, they felt that fatigue was a major barrier to enjoying these activities. Feelings of boredom were frequent, often because participants felt limited to a few activities, which became repetitive and therefore not enjoyable.

*“Um, now cause obviously I can only really like, go to school and like going out actually. . .So I just get a bit like bored like around the house.”* (P13, 13-year-old female)

*“I wouldn’t say it was fun no but, no I just do it.”* (P11, 17-year-old female)

#### Curtails socialising

Socialising with friends and peers was curtailed by CFS/ME. Generally, the duration and frequency of time spent with friends had decreased due to CFS/ME symptoms. For some, this was the result of not attending school, which left them with online communication, such as texting, as their only means of connecting socially. Others felt that when they were invited to see friends, a lack of motivation got in the way.

*“. . .sort of play games with my friends, online games, I don’t really get out the house much, I’m not really able to.”* (P10, 15-year-old male)

*“Um, probably when like - because I used to be really like up for it and like we have like a group chat and people would be like ‘Oh do you want to meet today’ and everyone would say yes, I’d always be like ‘Oh god like no I cannot be bothered’. But I did used to like really want to go out and do things like that.”* (P7, 14-year-old female)

Friendships were affected by the illness, and this limited opportunities to engage in potentially enjoyable shared activities.

*“I lost a lot of friends so, through being ill cause of chronic fatigue. Um, so I don’t have any friends to do anything thing with”* (P11, 17-year-old female)

Parents reflected on the vicious cycle this created, reinforcing low mood.

*“and you-you don’t see your friends. . .that’s a vicious circle because she knows it would lift her mood to see them but she’s not feeling up to it, she’s got-she’s got one good friend that she’s sees occasionally”* (parent of P14, 13-year-old female)

Not being able to take part in social life was experienced as ‘watching from afar’ (P9, 16-year-old female) and negatively impacted normal developmental processes.

*“. . .cause (interviewee name)’s sixteen, she’s becoming a woman, she’s seeing friends, um, having sixteenth birthdays parties. . .and she-she’s left out that important right of passage really.”* (parent of P9, 16-year-old female)

*Comparison of the experience of anhedonia in those who were depressed to those who were not depressed.*

Of those who scored threshold for anhedonia, 18 (72%) met the full major depressive disorder DSM-5 diagnostic criteria. To explore the experience of anhedonia further, we compared the narratives from participants who were depressed (interview recordings available for *n* = 16 of 18) to those participants who were not depressed (interview recordings available for *n* = 6 of 7). The themes were broadly similar. Both groups of participants talked about not being able to regularly do activities they used to due to their CFS/ME and about a lack of motivation and interest in activities. Participants who were depressed felt that a lack of motivation was constant whereas this occurred less frequently for those who were not depressed.

*“. . .sometimes I can’t be bothered like I know that I’ll like it, I just keep trying”* (P3, 17-year-old female, not depressed).

*“. . .just can’t get any motivation to do writing or college work. I think it’s just because I’m more tired than usual that I just don’t want to do it.”* (P1, 16-year-old female, depressed).

Participants who were not depressed talked about still experiencing some enjoyment once they had started an activity or believed they would despite a lack of motivation, whereas this was not evident in the accounts of those who were depressed.

“*I’ve definitely lost interest in it a tiny bit but not so much that I don’t want to do it.*” (P4, 13-year-old male, not depressed).

*“It’s usually fun, it’s before I’m going out that I don’t really want to because I don’t think it’s going to be fun”* (P2, 14-year-old male, not depressed)

Participants who were not depressed also appeared more optimistic and hopeful about activities and the ability to do activities in the future. This enthusiasm did not come across with participants who were depressed.

*“I’m just kind of like, looking forward to when I feel a bit better and can go out. . .I’ve been like feeling like a bit better and can like plan and stuff so like it’s kind of gotten better so I know that I can look forward to going out”* (P13, 13-year-old female, not depressed)

*“Um, probably when like - because I used to be really like up for it and like we have like a group chat and people would be like ‘Oh do you want to meet today’ and everyone would say yes, I’d always be like ‘Oh god like no I cannot be bothered’. But I did used to like really want to go out and do things like that.”* (P7, 14-year-old female, depressed)

*“I don’t really have the motivation to see them [friends] outside of college.”* (P1, 16-year-old female, depressed)

## Discussion

There has been little previous attention given to the experience of anhedonia in adolescents with CFS/ME. In our sample of 164 adolescents with CFS/ME, a substantial minority (42%) were experiencing some anhedonia, one third of whom had clinically significant anhedonia. This means that one in eight adolescents with CFS/ME had clinically significant anhedonia. Those who had clinically significant anhedonia were not necessarily more functionally impaired or fatigued, but they scored higher on both anxiety and depression questionnaires. In depth qualitative analysis found that participants described having lost interest and motivation in doing activities they had previously enjoyed. When they do activities, CFS/ME gets in the way of enjoyment, as it curtails their ability to do the activities which they previously enjoyed, and new activities felt like a poor substitute. Participants also talked about the negative thoughts they had about activities, for example that they would not enjoy activities, or they were ‘left out’ from the things that their peers were able to do. Nearly three quarters of the adolescents who had clinically significant anhedonia met the criteria for a diagnosis of major depressive disorder, for which anhedonia is a core symptom. The narratives describing anhedonia were similar for those who were depressed and those who had clinically significant anhedonia but were not depressed, although the accounts of those who were depressed used more negative language about the lack of pleasure derived from activities.

We found that anhedonia is common in adolescents with CFS/ME, and not just among those who are depressed. The rate of anhedonia amongst adolescents with CFS/ME and depression is comparable to that in adolescents with major depressive disorder presenting to mental health services. For example, one previous study reported that approximately two thirds were experiencing threshold anhedonia, using the same diagnostic interview that we used in the current study (Goodyer et al., 2017). This is unsurprising in the mental health population, given that anhedonia is one of the core symptoms required to meet the diagnostic criteria for major depressive disorder (APA, 2013). However, the prominence of anhedonia in this sample, even in those who did not meet diagnostic criteria for depression, is more remarkable, and the narratives of those adolescents with anhedonia but not ‘major depressive disorder’ did indicate similarities in the symptom experience of anhedonia and it was generally described as problematic and undesirable. This highlights the need to explore anhedonia further as a symptom in this population. We found that those with clinically significant anhedonia had raised scores on both anxiety and depression using a screening questionnaire. We also found that the accounts of those who were depressed were generally more negative and extreme, which may reflect the negative, global and distorted cognitive biases typical of depression (Schwartz & Maric, 2015; Weeks et al., 2017), including in adolescents with CFS/ME who have co-morbid psychopathology (Loades et al., 2020a). However, there were more similarities than differences in the experience of anhedonia described by those who did and did not meet the diagnostic criteria for depression. It may be that those who did not meet the full diagnostic criteria for depression were experiencing subthreshold symptoms of depression in addition to clinically significant anhedonia. They may therefore be at risk of becoming depressed, and future studies could examine this using a longitudinal approach.

In the current study, CFS/ME was experienced by participants as a barrier to engaging in activities which reduced or hindered their consummatory pleasure (i.e. their enjoyment whilst actually doing the activity). This is consistent with previous findings that low mood and negative appraisals of the limitations of CFS/ME inhibited enjoyment of activities in adolescents with comorbid depression and CFS/ME (Taylor et al., 2017). The lack of enjoyment in activities, of which they have limited choice of, appears to be a significant factor in the development of depression in this population.

CFS/ME also seemed to affect motivation to engage in activity, impacting on anticipatory pleasure. This is comparable to findings of the experience of anhedonia in a non-CFS/ME population where participants reported a willingness to exert effort but a lack of drive for general activities (Watson et al., 2020). These participants also reported experiencing low energy and fatigue which was linked to their lack of motivation to engage in activity. This would indicate that the fatigue of CFS/ME may be a risk factor for the reduction of anticipatory pleasure, which could lead to developing co-morbid depression. Watson et al. (2020) also reported that adolescents with anhedonia felt socially detached which correlates with the theme of social activity where participants reported that their low mood was exacerbated by social withdrawal and the limitations of not being a ‘normal’ teenager.

It may be that a vicious cycle serves to maintain anhedonia in adolescents with CFS/ME. CFS/ME results in considerable functional limitations (Crawley & Sterne, 2009; Kennedy et al., 2010; Smith et al., 2003). Over time, these limitations may impact on mood, motivation and interest, resulting in a cycle where affected individuals lack enjoyment of activities due to CFS/ME symptoms and also lack the motivation to engage in the activities, further compounding their difficulties. Additionally, adolescents with CFS/ME may miss the enjoyable aspects of activities when they are unable to complete them, which could be detrimental to their mood.

## Strengths and limitations of the current study

A strength of the present study is that a large sample was used to obtain prevalence of anhedonia and depression in CFS/ME using a gold standard diagnostic interview. Furthermore, this is the first study to focus on anhedonia in the adolescent CFS/ME population. However, the main limitation to this study is that the interviews, which were analysed thematically, were part of a semi-structured diagnostic interview, and therefore not designed for generating rich qualitative data or probing for meaning beyond the level of detail required for the interviewer to make a judgement about the presence of a symptom and to rate the severity of it. Future studies could set out to explore anhedonia specifically, using an interview schedule designed specifically to explore the experience of anhedonia to gain further insight into the experience of this symptom in this population. Another limitation is that the interviews were conducted within 8 weeks of an initial appointment with a specialist CFS/ME service, meaning that the experiences of adolescents at different stages of their illness trajectory were not necessarily captured, although the illness duration varied considerably amongst those included in this study.

## Clinical and research implications

The present study suggests a need to focus on the anticipatory and consummatory pleasure of adolescents with CFS/ME and suggests that, at least for a substantial minority, a focus on increasing positive affect might be important. This is consistent with a perspective of anhedonia as a dimensional loss of positive affect (De Fruyt et al., 2020). Current treatments for adolescent CFS/ME include: activity management, cognitive behavioural therapy, graded exercise therapy (Brigden et al., 2017). All these approaches involve engaging in activity, and anhedonia may impact on this. Previous qualitative research with adolescents with co-morbid CFS/ME and depression concluded that there were mixed opinions about the usefulness of cognitive behavioural therapy but that activity management helped to increase anticipatory pleasure for activities by giving participants activities to look forward to (Taylor et al., 2017). A cognitive behavioural therapy approach in adults with depression and anhedonia, which focuses on increasing the positive valence system as well as employing the traditional cognitive behavioural treatment strategies which emphasise decreasing the negative affect system, has shown initial promise (Dunn et al., 2019). Elements of this approach may prove to be useful for those adolescents with CFS/ME who are struggling with anhedonia.

Future research could investigate how professionals can best assess anhedonia in adolescents with CFS/ME, including what measures can be used to screen for anhedonia and specific indicators that can be identified at assessments. Given that McMakin et al. (2012) reported poorer treatment outcomes for depressed youth with presenting anhedonia, it is possible that anhedonia may impact on treatment outcomes. Longitudinal studies could be used to investigate whether anhedonia impacts on treatment outcome. It would also be valuable to establish what impact current treatments for CFS/ME have on anhedonia, and to explore how treatments might need to be adapted for those with anhedonia.

## Conclusion

This study highlights the prevalence and experience of adolescents with comorbid CFS/ME and depression. Forty-two percent of participants experienced some anhedonia and one in eight had clinically significant anhedonia. Participants reported that the impact of CFS/ME affected both their consummatory pleasure and anticipatory pleasure of activities. A vicious cycle appears where adolescents do not enjoy activities due to symptoms of CFS/ME and thus lose motivation to do them. These findings indicate that treatment for comorbid depression and CFS/ME should focus on anhedonia symptoms and the possibility of treatments which aim to increase positive affect. Further research could be done using qualitative assessment designed for rich data to gain a wider scope of experience of this symptom of depression.
